# The Oxidative–Mitochondrial–Inflammatory Axis in Retinitis Pigmentosa: Extracellular mtDNA as Biomarker and Therapeutic Read-Out

**DOI:** 10.3390/antiox15091110

**Published:** 2026-09-02

**Authors:** Rossella Grimaldi, Francesca Franco, Enzo Maria Vingolo

**Affiliations:** Sense Organs Department, UOSD of Ophthalmology, Sapienza University of Rome, Polo Pontino-Ospedale A. Fiorini, 04019 Terracina, Italy; rossella.grimaldi@uniroma1.it (R.G.); f.franco@uniroma1.it (F.F.)

**Keywords:** retinitis pigmentosa, oxidative stress, mitochondrial DNA, cGAS–STING, NLRP3 inflammasome, extracellular vesicles, liquid biopsy, biomarker, antioxidants, neuroinflammation

## Abstract

Retinitis pigmentosa (RP) is the most common inherited retinal dystrophy (prevalence ~1:4000) and a leading Mendelian cause of working-age blindness. Despite marked genetic heterogeneity, its progression converges on a common secondary cascade of outer-retinal hyperoxia, increased reactive oxygen species (ROS), and mitochondrial dysfunction that drives cone degeneration and central vision loss. Because this oxidative cascade is largely genotype-independent and pharmacologically tractable, oxidative stress is a cross-cutting therapeutic target. Within it, mitochondrial DNA (mtDNA) is a key element: once released from damaged photoreceptors—free or within exosomes—it may act as a damage-associated molecular pattern (DAMP), engaging TLR9, cGAS–STING, and the NLRP3 inflammasome and sustaining chronic neuroinflammation. Extracellular mtDNA is therefore a potential integrative marker, simultaneously reflecting oxidative stress, mitochondrial dysfunction, cell death, and innate-immune activation. A central knowledge gap, however, remains: the mechanistic steps linking mtDNA to inflammation and to photoreceptor death have not been demonstrated in RP itself, and extracellular mtDNA has never been quantified in the ocular fluids of RP patients. In this review we appraise oxidative biomarkers in RP, propose extracellular mtDNA as a candidate biomarker of disease activity, and examine antioxidant and redox-modulating therapies—from N-acetylcysteine and elamipretide trials to DAMP-sensor inhibition—across experimental and clinical models. Finally, we propose extracellular mtDNA as a candidate pharmacodynamic endpoint and outline a path toward its validation.

## 1. Introduction

Retinitis pigmentosa (RP) is the most common inherited retinal dystrophy, with an estimated prevalence of approximately 1 in 4000 individuals and is the leading Mendelian cause of working-age blindness in the developed world [1,2]. It is a clinically and genetically heterogeneous group of rod–cone degenerations, with autosomal dominant, autosomal recessive, and X-linked inheritance and mutations in more than ninety genes governing phototransduction, intraflagellar transport, photoreceptor architecture, and energy metabolism [1,3]. Representative examples include *RHO* in dominant forms, *PDE6B*, *USH2A*, and *EYS* in recessive forms, and *RPGR* and *RP2* in X-linked forms [1,3]. Regardless of the initiating mutation, the shared consequence is rod photoreceptor death, which accounts for the early signs of nyctalopia and mid-peripheral ring scotomas [1,2]. The loss of most rods triggers a common pathogenic cascade characterized by oxidative stress, mitochondrial dysfunction, and neuroinflammation, with progressive cone degeneration and consequent constriction of the visual field up to central vision loss [4,5,6,7].

This convergence has the following decisive therapeutic implication: whereas gene-specific correction can benefit only a minority of patients, the secondary cascade is a common, downstream, and pharmacologically accessible target shared across the entire disease.

Within this cascade, mitochondrial DNA (mtDNA) occupies a position of particular interest because it is not merely a marker of oxidative mitochondrial damage but, once released from damaged photoreceptors, free or packaged in exosomes, it behaves as a damage-associated molecular pattern (DAMP) that engages innate-immune sensors and perpetuates inflammation [8,9,10,11]. This dual identity positions mtDNA as a possible biomarker of disease activity in RP while simultaneously constituting a measurable molecular signature of the degenerative process.

**Figure 1:**
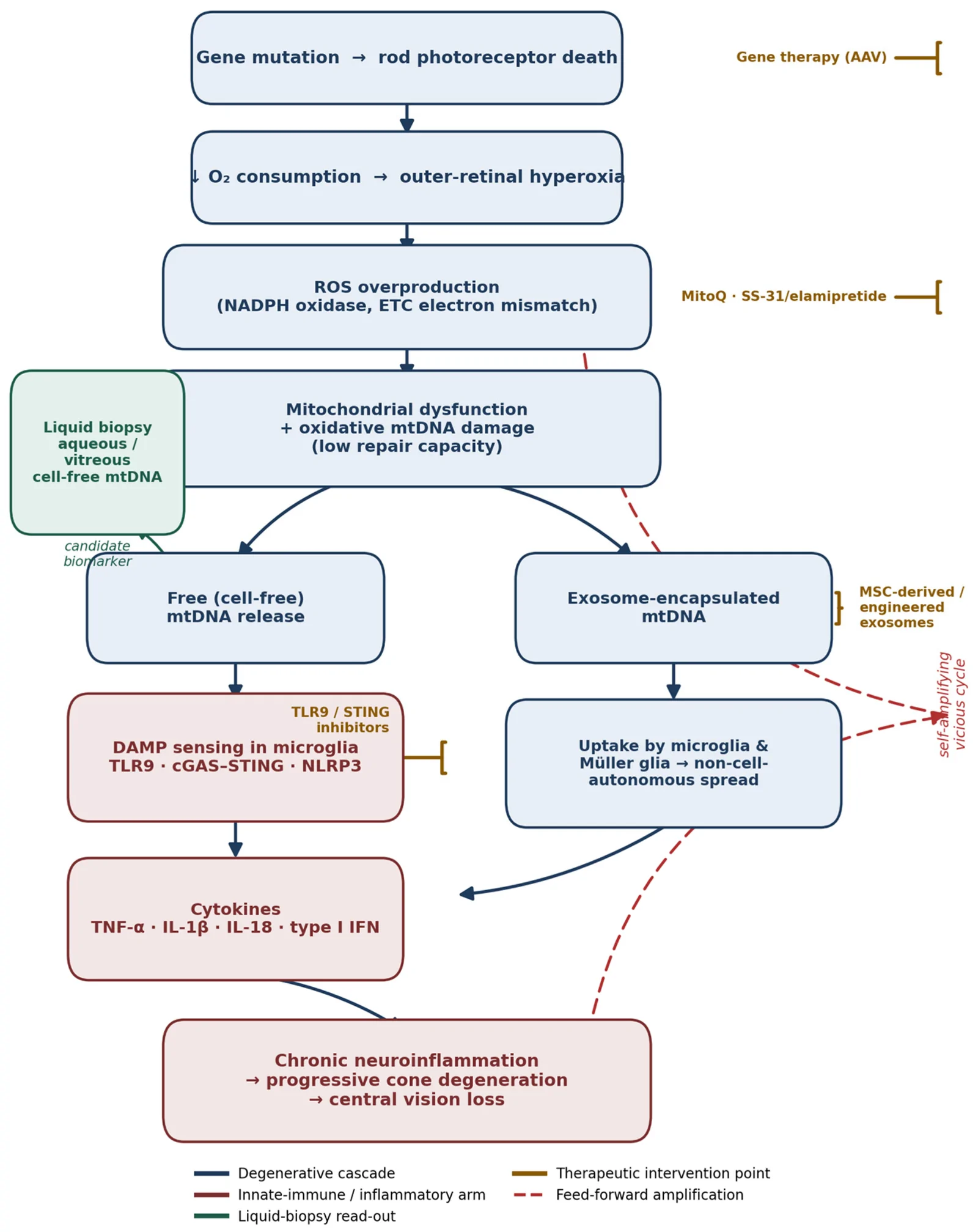
The self-amplifying oxidative–mitochondrial–inflammatory axis in RP. Rod loss drives outer-retinal hyperoxia, ROS, and mitochondrial dysfunction with mtDNA damage; released mtDNA (free and exosomal) engages TLR9, cGAS–STING, and NLRP3, sustaining neuroinflammation and cone loss. Therapeutic intervention points and the candidate cell-free mtDNA read-out are indicated. Solid arrows/boxes denote steps with direct RP evidence (human or animal RP); dashed elements (mtDNA–innate-immune engagement, vesicular propagation, and the cell-free read-out) are extrapolated from related retinal or non-ocular studies and not yet shown in RP. **Abbreviations:** AAV, adeno-associated virus; cGAS, cyclic GMP–AMP synthase; DAMP, damage-associated molecular pattern; ETC, electron transport chain; IFN, interferon; IL, interleukin; MSC, mesenchymal stem cell; mtDNA, mitochondrial DNA; NLRP3, NLR family pyrin domain-containing 3; ROS, reactive oxygen species; STING, stimulator of interferon genes; TLR9, Toll-like receptor 9; TNF-α, tumor necrosis factor α.

Consistent with the aims of this Special Issue, the present review (i) outlines the molecular mechanisms of the gene-independent oxidative cascade; (ii) identifies and appraises oxidative biomarkers, including the analytical conditions for measuring mtDNA in ocular fluids; and (iii) examines the efficacy of antioxidant and redox-modulating therapies across in vitro, in vivo, and clinical models, mapping them onto the nodes of the cascade and defining a validation agenda. Throughout, we grade the provenance of each line of evidence—human RP studies, RP animal models, related retinal diseases (AMD, diabetic retinopathy, and LHON), in vitro RP systems, and the broader non-ocular mitochondrial/inflammatory literature—so that mechanistic extrapolations are not mistaken for findings established in RP (Table 1 and Table 2, Figure 1). The present review is framed around this gap as follows: it assembles the mechanistic and cross-disease evidence for the axis, marks explicitly what remains unproven in RP, and defines the measurements that would close it.

## 2. The Gene-Independent Secondary Cascade: Hyperoxia, ROS, and Cone Death

Rod photoreceptors, which constitute roughly 95% of photoreceptors and are among the most oxygen-consuming cells in the body, govern the metabolic demand of the outer retina [4,12]. This demand depends on choroidal perfusion which, unlike the retinal vasculature, does not autoregulate in response to a falling demand. When rods degenerate, oxygen consumption by the outer retina collapses while choroidal supply continues, producing a relative tissue hyperoxia [4]. The excess oxygen fuels superoxide generation through electron-transport-chain imbalance and NADPH oxidase activation, overwhelming antioxidant defenses and generating further reactive species, including the highly damaging peroxynitrite [4,13].

The causal weight of this hypothesis is robustly and cross-species supported. In murine, porcine, and feline models of RP, oxidative-damage markers accumulate in cones after rod loss and—decisively—both antioxidant treatment and reduction in inspired oxygen to approximately 11% lower retinal superoxide and preserve cone structure and function [5,6,7,13,14]. These are interventional experiments that confirm the mechanism. On the human side, the evidence is convergent as follows: RP patients show impaired ocular antioxidant defense, with reduced total antioxidant capacity and extracellular superoxide-dismutase (SOD3) activity, increased thiobarbituric-acid reactive substances (TBARS, an index of lipid peroxidation), and activation of the nitric oxide pathway in aqueous humor and peripheral blood [12,15]. The link among rod loss, hyperoxia, oxidative damage, and cone death is therefore among the best characterized in inherited retinal dystrophies and identifies oxidative stress as a rational, genotype-independent therapeutic target. Together, these cross-species and human data establish oxidative stress as a necessary, genotype-independent mediator of cone death rather than an epiphenomenon.

## 3. Mitochondrial Dysfunction and mtDNA Vulnerability

Cones rely heavily on oxidative phosphorylation to sustain the metabolic cost of central and photopic vision, which makes their mitochondria both the principal source of ROS and one of its principal targets [4,16]. Oxidation of electron-transport-chain components disrupts electron flow, further increases mitochondrial ROS, and reduces ATP production, establishing a self-sustaining loop in which oxidative stress and mitochondrial dysfunction amplify one another. Mechanisms partly independent of ROS have also been documented, with direct impairment of phototransduction and retinal degeneration, underscoring the multifactorial nature of the mitochondrial contribution to RP [17]; consistently, interventions that normalize the mitochondrial redox state, such as photobiomodulation, are retinoprotective in RP models [18].

Mitochondrial DNA is particularly exposed within this loop: it lies in close proximity to the respiratory chain, is not protected by histones, and has limited repair capacity relative to nuclear DNA, so it readily accumulates oxidative lesions [16]. Because mtDNA encodes essential subunits of oxidative-phosphorylation machinery, such damage can compromise the expression of those very subunits, aggravating the energy deficit. Although genetically distinct from non-syndromic RP, the observation that primary mtDNA mutations cause syndromes with RP-like retinal degeneration, such as neuropathy, ataxia, and retinitis pigmentosa (NARP, m.8993T>G mutation in *MT-ATP6*) and MELAS (m.3243A>G), in which disrupted mitochondrial bioenergetics produces retinal and pigment-epithelium dystrophy [19,20], offers analogical support that mitochondrial bioenergetic failure alone can drive retinal and pigment-epithelium dystrophy; it does not, however, establish a role for mtDNA damage in non-syndromic RP. Moreover, oxidation of mtDNA may promote innate-immune activation [9].

### Mitochondrial Quality Control: Mitophagy as the Upstream Gatekeeper of mtDNA Release

Whether oxidatively damaged mtDNA is silently cleared or released to trigger inflammation is decided upstream by the efficiency of mitochondrial quality control (MQC), whose principal effector is mitophagy—the selective autophagic removal of damaged mitochondria. In the canonical ubiquitin-dependent pathway, loss of membrane potential stabilizes PINK1 on the outer mitochondrial membrane, which recruits and activates the E3 ligase Parkin; ubiquitinated mitochondria are then engulfed by autophagosomes and degraded in lysosomes, with receptor-mediated mitophagy (BNIP3, NIX/BNIP3L, and FUNDC1) providing a complementary, Parkin-independent route [21]. By degrading damaged organelles before their contents escape, mitophagy normally prevents oxidized mtDNA from reaching the cytosolic and extracellular compartments.

When MQC is overwhelmed or genetically impaired, this safeguard fails and mtDNA becomes an inflammatory trigger: in Parkin- or PINK1-deficient animals, mitochondrial stress leads to accumulation of mtDNA and activation of cGAS–STING, and the resulting inflammation and neurodegeneration are rescued by concurrent STING deletion, establishing mitophagy as a gatekeeper of the mtDNA–cGAS–STING/–NLRP3/–TLR9 axis. This checkpoint is directly relevant to the retina, where the retinal pigment epithelium and photoreceptors are mitochondria-rich and depend on efficient MQC, and where sustained oxidative stress dysregulates RPE mitophagy in AMD and diabetic-retinopathy models [21,22]. In RP, the chronic hyperoxia and ROS load that follow rod loss (Section 2) would be expected to overwhelm photoreceptor MQC, shifting the balance from degradation toward release of oxidized mtDNA; this upstream step, however, has not yet been directly demonstrated in RP and is inferred from related retinal disease and non-ocular models. Mitophagy enhancement (e.g., urolithin A, NAD^+^ precursors such as nicotinamide riboside, and AMPK activation) therefore represents an upstream therapeutic node complementary to the antioxidant and DAMP-sensor strategies of Section 7.

## 4. Released mtDNA as a DAMP: Amplification of Innate Immunity

Under sustained stress, damaged mtDNA can escape both from the organelle (into the cytosol) and from the cell (into the extracellular space), either free or within vesicles [8,11]. Because mtDNA is evolutionarily bacterial in character—circular, hypomethylated, and rich in unmethylated CpG motifs—it is read by the innate immune system as a danger signal through at least three convergent pathways.

Mechanistically, these are three distinct sensor systems. cGAS (cyclic GMP–AMP synthase) binds cytosolic double-stranded DNA in a largely sequence-independent manner and synthesizes the second messenger 2′3′-cGAMP, which activates the adaptor STING on the endoplasmic reticulum; STING recruits TBK1, which phosphorylates IRF3 to drive type I interferon transcription and additionally engages NF-κB [23]. Endosomal TLR9 recognizes unmethylated CpG motifs—abundant in the bacterially derived mtDNA—and signals through MyD88 to activate NF-κB and pro-inflammatory cytokine transcription [8]. The NLRP3 inflammasome requires the following two signals: a priming signal (NF-κB-dependent up-regulation of NLRP3 and pro-IL-1β) and an activation signal, delivered when oxidized mtDNA reaching the cytosol binds NLRP3 directly, nucleating assembly with the adaptor ASC and pro-caspase-1; active caspase-1 then matures IL-1β and IL-18 and cleaves gasdermin D, whose N-terminal fragment forms plasma-membrane pores [23,24,25]. A single molecular signal—released mtDNA—therefore feeds interferon, NF-κB, and inflammasome outputs simultaneously.

### 4.1. TLR9 and cGAS–STING

Endosomal TLR9, expressed by resident retinal microglia, recognizes CpG-rich mtDNA and activates NF-κB signaling, promoting the production of TNF-α and IL-1β; the release of mtDNA into the circulation has been identified as a TLR9 DAMP trigger in settings of tissue injury [8,9]. In parallel, cytosolic mtDNA is recognized by cyclic GMP–AMP synthase (cGAS), which generates 2′3′-cGAMP, activates STING and the TBK1–IRF3 axis, and induces type I interferons [11,26]. cGAS and STING are expressed in multiple retinal cell types, including photoreceptors, RPE, and microglia, and the pathway is demonstrably activated in dry AMD and diabetic retinopathy, where cytosolic DNA leakage in photoreceptors and RPE and the protective efficacy of STING inhibition—including BRD4 inhibition with JQ1—in reducing inflammation and degeneration have been documented [26,27,28]. The identification of released mtDNA as a TLR9/cGAS–STING trigger derives from non-ocular tissue injury [8,9,11]; direct retinal demonstration is so far confined to AMD and diabetic retinopathy [26,27,28]; neither pathway has been shown to be engaged by mtDNA in RP retina or in RP patients.

### 4.2. The NLRP3 Inflammasome and Inflammation in RP

The NLRP3 inflammasome responds to mitochondrial stress and oxidized mtDNA by activating caspase-1 and the maturation of IL-1β and IL-18 [10]. Unlike other nodes, here there is direct evidence in RP models: in canine models RP up-regulates NLRP3 inflammasome genes, and in the retinas of P23H mice (mutation of *RHO*) NLRP3 is detectable in cones and in roughly one-third of reactive microglia, with increased mature IL-1β and IL-18 and cleaved caspase-1 [29,30]. In human RP, corroboration is so far indirect (increased double-negative T lymphocytes in the aqueous humor [31]); the inflammasome has not been assayed in patient tissue, and extracellular mtDNA has not been shown to be its upstream trigger in genetically heterogeneous RP. NLRP3 inflammasome activation has been proposed to specifically mediate the bystander death of genetically normal cones in the P23H model, indicating a distinct neuroprotective target [29]. Consistently, ROS act as a secondary trigger of NLRP3, IL-1β, and IL-18 transcription in RP retinas [32], closing the loop between oxidative stress and inflammation.

### 4.3. Microglia, Müller Glia, and Neuroinflammation

Damaged or dying photoreceptors release signals that activate microglia and Müller glia, which transition toward pro-inflammatory phenotypes with secretion of TNF-α, IL-1β, IL-6, and IL-18 and amplify the damage [30,33]. In RP, microglia adopt a state of persistent inflammasome activation, and analysis of patients’ ocular fluids documents an active immune component as follows: CD3^+^CD4^−^CD8^−^ double-negative T lymphocytes are increased in the aqueous humor of RP patients, supporting a measurable inflammatory involvement in vivo [31]. Notably, the antioxidant N-acetylcysteine reduces NLRP3 expression and microglial infiltration by about 50% in P23H models, improving cone survival and function [30]—a direct example of how a redox intervention modulates inflammation downstream. Collectively, these pathways indicate that released mtDNA can convert mitochondrial injury into a self-sustaining inflammatory signal, although—as emphasized above—this full sequence is inferred rather than proven in RP.

### 4.4. From mtDNA to Cone Death: Pyroptosis, Ferroptosis, and Apoptosis

The pathological weight of these pathways lies in their convergence on photoreceptor death through several regulated modalities, so that released mtDNA is not only an inflammatory signal but also an index of ongoing cell death. Inflammasome activation couples mtDNA sensing directly to pyroptosis: caspase-1 cleaves gasdermin D to form membrane pores, releasing IL-1β and IL-18 and lysing the cell in a highly pro-inflammatory manner that amplifies surrounding injury [34,35]. In parallel, the same oxidative environment that damages mtDNA drives ferroptosis—an iron-dependent, lipid-peroxidation-driven death to which photoreceptors are especially prone given their polyunsaturated-fatty-acid-rich outer segments and high metabolic demand; iron chelation or augmentation of glutathione peroxidase 4 (GPX4) improves photoreceptor survival in RP and retinal-degeneration models [36,37]. Classical apoptosis, long recognized as the terminal route of rod and cone loss in RP, proceeds through mitochondrial outer-membrane permeabilization, which is itself a route of mtDNA egress [38], tying the death program back to the DAMP it liberates. These modalities are interconnected rather than mutually exclusive and share mitochondrial oxidative injury as their upstream node; consequently, extracellular mtDNA plausibly reports both inflammation and regulated cell death—the very property that motivates its use as an integrative read-out (Section 6 and Section 9). We note, however, that the specific contribution of each death modality in human RP remains to be quantified.

## 5. Free and Exosomal mtDNA: Propagation of Damage

Exosomes are small extracellular vesicles (approximately 30–150 nm) released through the endosomal system that carry proteins, lipids, mRNA, microRNA, and DNA fragments to recipient cells, where they can reprogram their function; they are a primary vehicle of intercellular communication [39]. This supports a functional division of labor between the two pools of extracellular mtDNA.

How mtDNA physically escapes the mitochondrion determines whether, and in what form, it reaches these sensors. Under apoptotic stress, oligomerized BAX and BAK form macropores in the outer mitochondrial membrane through which the inner membrane herniates, extruding matrix mtDNA into the cytosol [38]. Under sub-lethal oxidative stress, oligomerized voltage-dependent anion channels (VDACs) form comparable pores that release short, oxidized mtDNA fragments [40], while calcium overload and prolonged opening of the mitochondrial permeability transition pore (mPTP) permeabilize the inner membrane and further promote efflux [35]. Once cytosolic, mtDNA is either sensed directly (Section 4) or repackaged into exosomes and extracellular vesicles for export, providing a vehicle for intercellular transfer. In functional terms this release is not mere leakage but a danger signal—an evolutionarily conserved report of mitochondrial catastrophe that recruits innate immunity—so its physiological purpose and its pathological cost are two facets of the same event.

Free mtDNA is thought to act predominantly as an acute-injury DAMP and inflammatory trigger, whereas exosome-encapsulated mtDNA is proposed to serve as a vehicle for the non-cell-autonomous propagation of damage. By analogy with other tissues, vesicles released by stressed photoreceptors are proposed to be internalized by microglia and Müller glia, whose cargo can push these cells toward pro-inflammatory and pro-oxidant states. This non-cell-autonomous propagation is mechanistically plausible but, in RP, remains to be demonstrated directly; consistent with the principle that vesicular cargo mirrors the state of the retina, extracellular-vesicle RNA from *PRPF31*-mutant iPSC-derived RPE tracks the state of retinal degeneration in an RP-relevant system [41], currently the strongest RP-relevant but still indirect support. Taken together, these observations suggest that vesicular mtDNA could propagate damage beyond the initially affected photoreceptors, but direct demonstration in RP is still lacking.

## 6. Oxidative and Mitochondrial Biomarkers in RP

### 6.1. Established Oxidative Biomarkers

Several oxidative biomarkers are already used in RP research. Malondialdehyde (MDA) and TBARS report lipid peroxidation; 8-hydroxy-2′-deoxyguanosine (8-OHdG) reports oxidative DNA damage; and protein carbonyls and the reduced-to-oxidized glutathione ratio report protein oxidation and redox state, documented directly in the retinas and eyes of RP patients [4,15]. The study by Martínez-Fernández de la Cámara and colleagues in 56 patients and 60 controls demonstrated reduced ocular antioxidant capacity with lower SOD3 activity, increased TBARS, and up-regulation of the NO/cGMP pathway in aqueous humor, together with redox alterations in peripheral blood [12]; the BIRP observational case–control study is specifically dedicated to measuring such markers in the aqueous and plasma of RP patients [42]. These markers are informative, but each captures a single facet of the process and typically provides only a static snapshot. Aqueous humor and, increasingly, vitreous are accessible compartments for such measurements and reflect retinal pathology much as cerebrospinal fluid does in neurological disease [43].

### 6.2. Extracellular mtDNA as an Integrative Biomarker

Extracellular mtDNA is conceptually attractive because it is mechanistically integrative: a single analyte that could simultaneously reflect mitochondrial dysfunction, oxidative load, cell death, and innate-immune activation, and that might therefore possess superior construct validity as a measure of overall disease activity. It must be stated at the outset, however, that this proposal rests on indirect evidence and that extracellular mtDNA has not been measured in the ocular fluids of patients with RP. The supporting precedents come from other diseases and speak to plausibility and feasibility, not to RP-specific validity: in Leber hereditary optic neuropathy—a distinct, primary mitochondrial retinopathy—oxidative-stress and inflammatory biomarkers have been quantified in aqueous humor [44], demonstrating that molecular monitoring from ocular fluid is technically achievable, while in non-ocular neurodegenerative and critical illnesses (amyotrophic lateral sclerosis, Parkinson’s disease) cell-free mtDNA behaves as an informative biomarker [45,46]. These precedents establish biological plausibility and technical feasibility, but they do not demonstrate that extracellular mtDNA reflects disease activity in RP. Extracellular mtDNA therefore remains a proposed candidate biomarker, not an RP-specific or clinically validated marker. Establishing RP-specific validity would require its measurement in ocular fluids or plasma from well-characterized RP patients against appropriate controls, with correlation to genotype, disease stage, OCT structural metrics (e.g., ellipsoid-zone width), and visual function, and confirmation of longitudinal tracking of progression (Section 9).

### 6.3. Analytical Considerations

The credibility of mtDNA as a biomarker depends almost entirely on the rigor of measurement. Cell-free mtDNA is most robustly quantified by droplet digital PCR (ddPCR)—which provides absolute copy numbers, achieves limits of detection close to 1 copy/µL when targeting *ND1*, and is well suited to the low input typical of ocular fluids—or by quantitative PCR, targeting mitochondrial loci such as *ND1*, *ND4*, *COXIII*, or the D-loop, and expressing the result as the mtDNA/nuclear-DNA ratio relative to a single-copy reference gene (for example, *RNase P* or *B2M*) rather than as a raw concentration [45,47]. Fragment-length characterization is relevant, because a considerable proportion of plasma mtDNA circulates as very short fragments [48]. Pre-analytical control is decisive: standardized centrifugation to remove cells and platelets, prevention of hemolysis, and limitation of freeze–thaw cycles; in addition, the free fraction should be separated from the vesicle-encapsulated fraction (differential ultracentrifugation or size-exclusion chromatography) if the two pools are to be distinguished. The compartment matters too: aqueous humor, obtained by anterior-chamber paracentesis (~100–200 µL, outpatient, and repeatable), is the realistic route for longitudinal monitoring, whereas vitreous is richer but accessible mainly during vitrectomy. Finally, because circulating mtDNA increases with aging and systemic inflammation (“inflamm-aging”), ocular compartmentalization and appropriate normalization are validity conditions [49]. Quantification of extracellular mtDNA in the ocular fluids of RP patients has not yet been performed and represents the immediate priority of the field (Section 9). In sum, extracellular mtDNA is analytically measurable with existing digital-PCR methods but remains a candidate whose RP-specific validity must still be established; the quantification requirements summarized here define the analytical backbone of that validation (Section 9).

**Table 1:** Oxidative and mitochondrial biomarkers in RP (evidence-level column standardized).

	What it Reflects	Matrix/Method	Evidence Level (RP-Specific?)	Main Limitation
MDA/TBARS	Lipid peroxidation	Aqueous/serum; TBARS, HPLC	Human RP [12,15]	Single pathway; non-specific
8-OHdG	Oxidative DNA damage	Serum/urine; ELISA, LC-MS	Human RP, limited [15]	Single pathway; pre-analytical variability
Carbonyls/GSH:GSSG/SOD3	Protein oxidation, redox state, antioxidant defense	Tissue/aqueous; assays	Human RP [12,15]	Access; snapshot only
EV RNA (miRNA)	Cellular signaling cargo	Vitreous/aqueous; RT-qPCR, seq	In vitro RP model [41]	Complex isolation; not mtDNA
Cell-free/EV mtDNA	Integrated: mitochondrial dysfunction, oxidative load, cell death, innate immunity	Aqueous/vitreous; ddPCR, qPCR (mtDNA/nDNA)	No RP data—plausibility only: related retinal (AMD, DR, LHON) + non-ocular (ALS, PD) [44,45,46]	To be validated in RP; normalization

*Table 1 footnote—evidence-level vocabulary (add): human RP = direct study in RP patients; RP model = RP animal model;* in vitro *RP = RP-relevant cellular system; related retinal = other retinal disease (AMD, DR, LHON); non-ocular = the general/systemic mechanistic literature. Abbreviations: ddPCR, droplet digital PCR; DR, diabetic retinopathy; EV, extracellular vesicle; GSH:GSSG, reduced/oxidized glutathione ratio; LHON, Leber hereditary optic neuropathy; MDA, malondialdehyde; PD, Parkinson’s disease; ALS, amyotrophic lateral sclerosis; SOD3, extracellular superoxide dismutase; TBARS, thiobarbituric-acid reactive substances; 8-OHdG, 8-hydroxy-2′-deoxyguanosine*.

## 7. Antioxidant and Redox-Modulating Therapies

Because the secondary cascade is shared across genotypes, the therapies discussed below act on different nodes of this cascade as follows: upstream of mtDNA release, by reducing the oxidative damage that generates it (systemic and mitochondria-targeted antioxidants), and downstream, by blocking the sensors that mtDNA activates (inhibition of TLR9/cGAS–STING/NLRP3). This very shared location along the oxidative–mitochondrial–inflammatory axis makes extracellular mtDNA a candidate common pharmacodynamic endpoint, since it would allow monitoring of whether heterogeneous interventions are actually modulating the cascade, irrespective of the node on which they act. It must be stated from the outset, however, that the therapies do not relate to mtDNA in the same way: some should reduce its release by acting upstream, others neutralize its effects downstream, and still others—though effective—act through routes that mtDNA would not directly reflect. It is precisely this gradation that defines for which interventions extracellular mtDNA could serve as a response read-out. Table 2 maps each strategy to its cascade node and highest level of evidence; the agents listed there—vitamin A, docosahexaenoic acid, and lutein, N-acetylcysteine, MitoQ and elamipretide (SS-31), TLR9/cGAS–STING/NLRP3 inhibitors, mesenchymal-stem-cell exosomes, and AAV gene therapy—are each discussed in the subsections below.

### 7.1. Systemic Antioxidants: From Experimental Evidence to Clinical Trials in RP

In animal models, antioxidant cocktails, reduction in inspired oxygen, and NADPH-oxidase inhibition preserve cone structure and function [5,6,7,13,14], providing causal evidence that lowering the oxidative load slows degeneration. Clinically, the most extensive experience concerns nutritional supplementation: the randomized trial by Berson and colleagues of vitamin A palmitate (15,000 IU/day) and vitamin E suggested slowing the decline in ERG amplitude with vitamin A and a possible unfavorable effect of high-dose vitamin E [50]; subsequent studies evaluated the addition of docosahexaenoic acid (DHA) [51] and lutein [52], with marginal overall benefits on the primary endpoint and results limited to subgroup analyses. The DHAX trial in X-linked RP [53] and the Cochrane review [54] scaled back the magnitude of the effect, and a critical appraisal of the evidence highlighted the methodological limitations of these studies [55]. Balanced reading is that untargeted antioxidant supplementation produces, at best, a modest benefit, motivating more targeted strategies and more sensitive biomarkers to power trials.

A more targeted approach is N-acetylcysteine (NAC), which directly scavenges ROS and replenishes the glutathione pool [56]. NAC reduces oxidative damage and prolongs cone survival in RP models [57] and, in phase I of FIGHT-RP trial, oral NAC was shown to be safe and tolerated and improved cone function (visual acuity and macular sensitivity) in RP patients [58]; a phase III trial is ongoing. NAC exemplifies how a redox intervention can act upstream of both oxidative damage and downstream of mtDNA-dependent inflammation, reducing NLRP3 expression and microglial activation [30]. Because it acts upstream, reducing the ROS that damage and release mtDNA, NAC is an ideal candidate for testing the hypothesis: if the antioxidant effect is real, it should translate into a measurable reduction in ocular extracellular mtDNA, which would thereby become a response marker.

### 7.2. Mitochondria-Targeted Antioxidants

Acting at the source of ROS, MitoQ—a coenzyme-Q10 derivative conjugated to a lipophilic triphenylphosphonium cation—accumulates in the mitochondrial matrix in a membrane-potential-dependent manner, whereas SS-31 (elamipretide) binds cardiolipin in the inner mitochondrial membrane independently of the potential—thus reaching even depolarized mitochondria—stabilizing cristae, improving electron-transport efficiency, reducing ROS, and protecting mtDNA [59]. The ocular proof of concept is solid: elamipretide is neuroprotective for retinal ganglion cells in glaucoma models [60] and has been evaluated clinically in dry age-related macular degeneration in the ReCLAIM (phase 1) and ReCLAIM-2 (randomized phase 2) trials, with an acceptable safety profile and exploratory signals on low-luminance function and ellipsoid-zone preservation [61,62]. Although a dedicated RP study is still lacking, this class is biologically the most directly aligned with the mitochondria–mtDNA axis described here: by acting on mitochondrial integrity and directly protecting mtDNA, mitochondria-targeted antioxidants are the interventions for which a reduction in released extracellular mtDNA would be the most direct pharmacodynamic read-out.

### 7.3. Interrupting mtDNA-Dependent Inflammation

Targeting the DAMP-recognition node—TLR9 antagonism, cGAS–STING inhibition, or NLRP3 blockade—aims to sever the link between mitochondrial damage and chronic neuroinflammation. STING-directed approaches, including BRD4 inhibition (JQ1), are protective in oxidative models of retinal degeneration [28], and cGAS drives non-canonical inflammasome activation in AMD [27]; in RP, NLRP3 blockade has been proposed as a strategy against bystander cone death [29]. This direction is conceptually the most tightly coupled to the biomarker thesis: because these drugs block precisely the sensors (TLR9/cGAS–STING/NLRP3) that extracellular mtDNA activates, mtDNA would represent both the signal that justifies their use and the metric by which to verify their action upstream of the receptor.

### 7.4. Exosome-Based Therapies

Mesenchymal-stem-cell-derived extracellular vesicles (MSC-EVs) show anti-inflammatory, antioxidant, and neuroprotective effects in models of retinal degeneration [39,63]. In the *rd10* model of RP, MSC-EVs injected into the vitreous increase photoreceptor survival, preserve their structure, and improve visual function (optomotor and electroretinographic responses), suppressing the activation of microglia, Müller glia, and macrophages through axes such as miR-146a–*Nr4a3* and miR-21 [63,64]; in analogous models, MSC-derived exosomes delay photoreceptor degeneration by lowering IL-6, TNF-α, and IL-1β [65]. Engineered vesicles further offer a route for targeted delivery of antioxidant molecules or therapeutic RNAs across ocular barriers [39]. Compared with antioxidants, the link to mtDNA is here more indirect—the effect is predominantly anti-inflammatory and paracrine—but, by acting on neuroinflammation downstream of release, a decline in extracellular mtDNA and associated mediators could likewise signal efficacy for these therapies. These approaches, still early-stage and with challenges of production and standardization, engage the same exosomal biology implicated in the propagation of damage.

### 7.5. Gene Therapy

Gene supplementation by adeno-associated virus addresses the most upstream node. Voretigene neparvovec for biallelic *RPE65*-mediated inherited retinal dystrophy improved functional vision in a randomized phase 3 trial and is approved, demonstrating that AAV therapy can be disease-modifying within the RP spectrum [66]. Unlike redox strategies, however, gene therapy does not act on the oxidative cascade but upstream of it, on the mutation: extracellular mtDNA would therefore not be a direct mechanistic endpoint, at most a non-specific indicator of reduced overall cell death. It also remains genotype-specific, whereas redox- and inflammation-directed strategies remain complementary and broadly applicable. Its relevance to the present axis is indirect but mechanistically clear: by restoring the missing gene product and preserving photoreceptors, gene supplementation removes the upstream driver of hyperoxia, ROS, and mitochondrial injury, and thereby reduces the oxidative damage and release of mtDNA that fuel the downstream inflammatory and cell-death cascade. Gene therapy thus acts at the origin of the axis, whereas antioxidant, DAMP-directed, and exosome strategies act on its consequences; its principal limitation is that it is mutation-specific and currently available for only a minority of genotypes.

Table 2 summarizes these strategies by cascade node and by available level of evidence. Overall, the therapeutic landscape spans validated antioxidant trials and still-preclinical DAMP-directed and exosome strategies, unified by their action on the shared oxidative–mitochondrial–inflammatory cascade.

**Table 2:** Redox-modulating therapies (evidence-level column standardized).

Strategy	Target Node	Representative Agents	Highest Level of Evidence	Main Limitation
Systemic antioxidants	ROS/oxidative load	Vitamin A; DHA; lutein; NAC	Human RP (RCTs; FIGHT-RP) [50,51,52,53,54,55,58]	Modest/heterogeneous effect
NOX inhibition/cocktail	ROS generation	NOX inhibitors; cocktail; 11% O_2_	RP models [5,6,7,13,14]	Limited human translation
Mitochondria-targeted antioxidants	ROS at source/mtDNA	MitoQ; SS-31 (elamipretide)	Related retinal (AMD, ReCLAIM); no RP trial [59,60,61,62]	No trial in RP
DAMP-sensor inhibition	TLR9/cGAS-STING/NLRP3	STING/BRD4 (JQ1); anti-NLRP3	RP + AMD models; no RP human data [27,28,29]	Preclinical in RP
Exosomes (MSC/engineered)	Glial reprogramming/release	MSC-EVs; engineered EVs	RP model (*rd10*) [63,64,65]	Production, standardization
Gene therapy (AAV)	Upstream mutation	Voretigene neparvovec (*RPE65*)	Approved; *RPE65* subtype only [66]	Genotype-specific

*Table 2 footnote—same evidence-level vocabulary as Table 1. Abbreviations: AAV, adeno-associated virus; DHA, docosahexaenoic acid; EV, extracellular vesicle; MSC, mesenchymal stem cell; NAC, N-acetylcysteine; NOX, NADPH oxidase; RCT, randomized controlled trial*.

## 8. Discussion

The literature reviewed here converges on a coherent picture: in RP, oxidative stress, mitochondrial dysfunction, and innate-immune activation form a single self-amplifying cascade rather than parallel processes, with extracellular mtDNA at its center as both an effector and a measurable output. The principal strength of this framing is integrative: unlike single-pathway markers such as TBARS or 8-OHdG, a cell-free mtDNA read-out could in principle capture several mechanistic axes at once and track them longitudinally from an accessible ocular compartment.

A legitimate question is what a molecular mtDNA read-out could add to the biomarkers already used in RP. Current outcome measures are predominantly downstream and structural: the ellipsoid-zone (EZ) width on SD-OCT—the most sensitive validated structural marker—constricts by roughly 100–150 µm per year [67,68]; the hyperautofluorescent ring on fundus autofluorescence contracts by ~0.5 mm^2^ per year and correlates with EZ width and visual-field loss [68]; and the full-field ERG, although the reference functional test, is frequently reduced to non-detectable levels early in RP, producing a floor effect that limits its sensitivity to further change. These measures faithfully quantify photoreceptor loss that has already occurred, but they change slowly and capture the consequence rather than the ongoing activity of disease. Extracellular mtDNA is conceptually complementary: as a molecular index of active mitochondrial injury, oxidative load, and innate-immune engagement, it could in principle report disease activity in real time—potentially changing before irreversible structural loss and serving as a pharmacodynamic marker of target engagement, including where the ERG floor precludes functional monitoring. We stress that this added value is proposed rather than demonstrated and must be established empirically against these established biomarkers (Section 9).

Several limitations temper this proposal. First, the direct evidence base in RP is still incomplete: while mtDNA-driven engagement of TLR9, cGAS–STING, and NLRP3 is documented in related retinal diseases and in animal models, extracellular mtDNA has not yet been quantified in the ocular fluids of RP patients, so its association with disease stage and activity is currently inferred rather than demonstrated. Second, mtDNA quantification is highly sensitive to pre-analytical and normalization choices, and circulating mtDNA is confounded by aging and systemic inflammation, making ocular compartmentalization and standardized assays prerequisites for any clinical use. Third, the therapeutic links are heterogeneous in strength: redox- and DAMP-directed interventions map naturally onto an mtDNA read-out, whereas gene therapy does not. Rather than undermining the rationale, these caveats define the validation agenda set out in Section 9, specifying exactly which measurements would confirm—or refute—the proposed biomarker.

## 9. Future Perspectives and Validation Agenda

To move extracellular mtDNA from a mechanistically grounded concept to a clinical tool in RP, the following specific and directly testable studies are needed:

1. A case–control quantification of cell-free and exosomal mtDNA (mtDNA/nDNA by ddPCR on *ND1* and *ND4*) in the aqueous humor of genetically characterized RP patients versus controls, stratified by genotype and stage (ellipsoid-zone width on OCT, visual-field index, full-field stimulus threshold).
2. Longitudinal correlation of ocular cell-free mtDNA with structural and functional progression, to test whether it tracks disease activity in the individual patient.
3. Demonstration, in human RP tissue or microglia, of mtDNA engagement of TLR9, cGAS–STING, and NLRP3, extending to RP what is already documented in models and related diseases.
4. Quantification of the free–exosomal partition and of mtDNA transfer to microglia and Müller glia in human or non-human-primate retina.
5. Definition of pre-analytical and normalization standards for ocular cell-free mtDNA and of the relationship between ocular and plasma compartments.
6. An interventional proof of pharmacodynamic value: in RP models, testing whether antioxidant therapy (for example, NAC or elamipretide) or DAMP-sensor inhibition reduces ocular cell-free mtDNA in parallel with cone preservation.
7. Head-to-head comparison of extracellular mtDNA with established structural and functional biomarkers (EZ width, FAF ring area, full-field and multifocal ERG, microperimetry) to determine its added value for disease staging, prognosis, and therapeutic monitoring in RP.
8. Determination of whether extracellular mtDNA release in RP reflects inflammation alone or also ongoing regulated cell death (pyroptosis, ferroptosis, and apoptosis), by correlating ocular mtDNA with cell-death and lipid-peroxidation markers.

## 10. Conclusions

In retinitis pigmentosa, the decisive and pharmacologically tractable therapeutic target is not the single mutation but the secondary cascade—hyperoxia, oxidative stress, mitochondrial dysfunction, and neuroinflammation—on which the various genotypes converge. Within this cascade, extracellular mitochondrial DNA occupies a peculiar position: it is simultaneously an effector, fueling neuroinflammation as a DAMP, and a read-out, since it integrates into a single measurable molecule the oxidative load, mitochondrial dysfunction, cell death, and innate-immune activation. From this dual nature follows the operational proposal of this review: extracellular mtDNA is not so much a new target as a new way of measuring whether the known targets are being hit. The redox and anti-inflammatory strategies appraised here—from systemic antioxidants such as N-acetylcysteine, to mitochondrial antioxidants such as elamipretide, to inhibition of DAMP sensors—all act on this cascade and could share a single pharmacodynamic endpoint in extracellular mtDNA. The priority, therefore, is its validation: quantifying it in the ocular fluids of RP patients, with appropriate normalization, and qualifying it as a marker of disease activity and treatment response. Only then can a solid mechanistic rationale translate into a clinical tool capable of guiding and measuring therapy across the entire genetic spectrum of the disease.

## Data Availability

No new data were created or analyzed in this study. Data sharing is not applicable to this article.

## Abbreviations

8-OHdG, 8-hydroxy-2′-deoxyguanosine; AAV, adeno-associated virus; AMD, age-related macular degeneration; cGAS, cyclic GMP–AMP synthase; DAMP, damage-associated molecular pattern; ddPCR, droplet digital PCR; DHA, docosahexaenoic acid; DR, diabetic retinopathy; ERG, electroretinogram; EV, extracellular vesicle; GSH:GSSG, reduced/oxidized glutathione ratio; IRD, inherited retinal dystrophy; LHON, Leber hereditary optic neuropathy; MDA, malondialdehyde; MELAS, mitochondrial encephalomyopathy, lactic acidosis, and stroke-like episodes; MSC, mesenchymal stem cell; MSC-EV, mesenchymal-stem-cell-derived extracellular vesicle; mtDNA, mitochondrial DNA; NAC, N-acetylcysteine; NARP, neuropathy, ataxia, and retinitis pigmentosa; nDNA, nuclear DNA; NF-κB, nuclear factor κB; NLRP3, NOD-, LRR- and pyrin domain-containing protein 3; NO, nitric oxide; NOX, NADPH oxidase; OCT, optical coherence tomography; ROS, reactive oxygen species; RP, retinitis pigmentosa; RPE, retinal pigment epithelium; SOD3, extracellular superoxide dismutase; STING, stimulator of interferon genes; TBARS, thiobarbituric-acid reactive substances; TBK1, TANK-binding kinase 1; TLR9, Toll-like receptor 9; TNF-α, tumor necrosis factor α. ASC, apoptosis-associated speck-like protein containing a CARD; BAX/BAK, Bcl-2-associated X protein/Bcl-2 antagonist killer; EZ, ellipsoid zone; FAF, fundus autofluorescence; GPX4, glutathione peroxidase 4; GSDMD, gasdermin D; IRF3, interferon regulatory factor 3; MQC, mitochondrial quality control; mPTP, mitochondrial permeability transition pore; TBK1, TANK-binding kinase 1; VDAC, voltage-dependent anion channel.
